# Quantitative analysis of pertussis, tetanus, and diphtheria antibodies in sera and breast milk from Tdap vaccinated women using a qualified multiplex assay

**DOI:** 10.1128/msphere.00527-23

**Published:** 2024-03-18

**Authors:** Susana Portillo, Jennifer Oshinsky, Margaret Williams, Sandra Yoder, Yuanyuan Liang, James D. Campbell, Miriam K. Laufer, Kathleen M. Neuzil, Kathryn M. Edwards, Marcela F. Pasetti

**Affiliations:** 1 Center for Vaccine Development and Global Health, University of Maryland School of Medicine, Baltimore, Maryland, USA; 2 Department of Pediatrics, University of Maryland School of Medicine, Baltimore, Maryland, USA; 3 Division of Infectious Diseases, Department of Pediatrics, Vanderbilt University Medical Center, Nashville, Tennessee, USA; 4 Department of Epidemiology and Public Health, University of Maryland School of Medicine, Baltimore, Maryland, USA; Weill Cornell Medicine, New York, New York, USA

**Keywords:** pertussis, Tdap, maternal vaccines, multiplex, infant immunity, infant vaccines

## Abstract

Pertussis (whooping cough) is a reemergent, highly contagious respiratory infection of public health concern. Infants prior to initiation of their primary vaccination series are the most vulnerable to severe infection, and even death. Vaccination during pregnancy is an efficacious means of reducing infection in infants. This approach relies on boosting maternal immunity and passive transfer of antibodies to the infant via placenta and breast milk. Similarly, maternal vaccination post-partum can enhance maternal-infant immunity. To support the analysis of pertussis immunity in the context of maternal-infant immunization, we developed a high throughput multiplex assay for simultaneous quantification of serum IgG antibodies against pertussis vaccine antigens: pertussis toxin (PT), filamentous hemagglutinin (FHA), pertactin (PRN), and fimbriae (FIM2/3), and against tetanus (TT) and diphtheria toxoids (DT), using the Meso Scale Discovery (MSD) platform. The assay was qualified, and specificity, sensitivity, accuracy, precision, linearity, and robustness were demonstrated. The assay was subsequently adapted for quantification of IgG and IgA in breast milk. Applied to a serological survey of pregnant women living in the United States and sub-Saharan Africa, this method revealed differences in magnitude and breadth of antibody profile, consistent with history of vaccination. A longitudinal analysis of Tdap responses in women vaccinated post-partum demonstrated a rapid increase in serum IgG that remained elevated for up to 24 months. Likewise, high levels of vaccine-specific IgA and IgG antibodies were present in breast milk, although they exhibited faster decay. This multiplex MSD assay is a reliable and practical tool for quantification of pertussis, tetanus, and diphtheria antibodies in serum and breast milk in serosurveys or vaccine studies.

**IMPORTANCE:**

Pertussis (whooping cough) has reemerged in recent years. Vaccination during pregnancy is an effective approach to prevent illness during the first months of life. We developed a multiplex assay for quantification of pertussis, tetanus, and diphtheria serum antibodies using the Meso Scale Discovery (MSD) platform; the method was qualified, and specificity, precision, accuracy, linearity, and limits of quantification were defined. It was also adapted for quantification of antibodies in breast milk. We successfully determined serostatus in women from different regions and with different vaccination histories, as well as responses to Tdap in blood and breast milk post-partum. This is the first description of a multiplex assay for the quantification of pertussis, tetanus, and diphtheria antibodies in breast milk.

## INTRODUCTION

Pertussis (or whooping cough) is a severe and sometimes fatal respiratory infection. The most vulnerable group is infants under 3 months of age who have not yet begun their primary vaccination series and have scant to no immunity (1). Despite high rates of routine immunization, the incidence of whooping cough has increased in recent years; this has been attributed to several factors, including short-lived immunity elicited by the acellular pertussis vaccine presently in use in high-income countries. Maternal immunization has been recommended in industrialized countries to boost maternal immunity and ensure that sufficient levels of antibodies are transferred to the infant via placenta and breast milk to shield them from infection until they begin their primary vaccination series.

Practical and reliable methods to assess pertussis immunity and responses to vaccination are greatly needed. Commercial serologic assays, typically used for diagnosis, lack information on assay components and performance, and do not meet the quality standards required for clinical study end point data. High throughput technologies for quantification of antibodies are attractive for expediency and conservation of specimens. There is special interest in practical methods suitable for the quantification of antibodies in different matrices, including mucosal secretions. Analysis of pertussis immunity in breast milk from mothers vaccinated during pregnancy or perinatally is important as maternal milk not only promotes infant growth but also contains a variety of immunological effectors, most importantly antibodies directed against potential pathogens. Features of human mucosal immunity against pertussis remain largely unknown. Antibodies in breast milk have been examined with limited scope (2–4).

Bioanalytical tools to support clinical study endpoints require rigorous development and evaluation of performance. Herein we describe the development and qualification of a multiplex immunoassay for quantification of pertussis, tetanus, and diphtheria serum IgG using the Meso Scale Diagnostics (MSD) technology (5). The principle of this assay remains the same as the indirect enzyme-linked immunosorbent assay (ELISA), except that multiple antigens are immobilized on spatially distinct spots within a single well of a 96-well plate, and each spot produces a unique signal, allowing for simultaneous detection of multiple antibody specificities in a single test: tetanus (TT) and diphtheria toxoid (DT) as well as *B. pertussis* toxin (PT), filamentous hemagglutinin (FHA), pertactin (PRN), and fimbriae (FIM2/3) were included in our assay plates. The analytical assay qualification involved analysis of specificity, relative accuracy, and precision, dilutional linearity and parallelism, lower limits of quantification, and robustness. This multiplex assay was further adapted for quantification of IgG and IgA in breast milk.

In addition to qualifying the multiplex assay and confirming its suitability for the intended purpose, we determined the antibody profile (serostatus) in women living in the United States who received Tdap during pregnancy and in women living in Malawi who did not receive pertussis vaccine during pregnancy. We also examined antibody responses to Tdap in sera and breast milk from U.S. women vaccinated post-partum (at delivery) and monitored for antibody persistence.

## RESULTS

### Multiplex assay qualification

#### Assay format and in-house standard calibration

**Fig 1:**
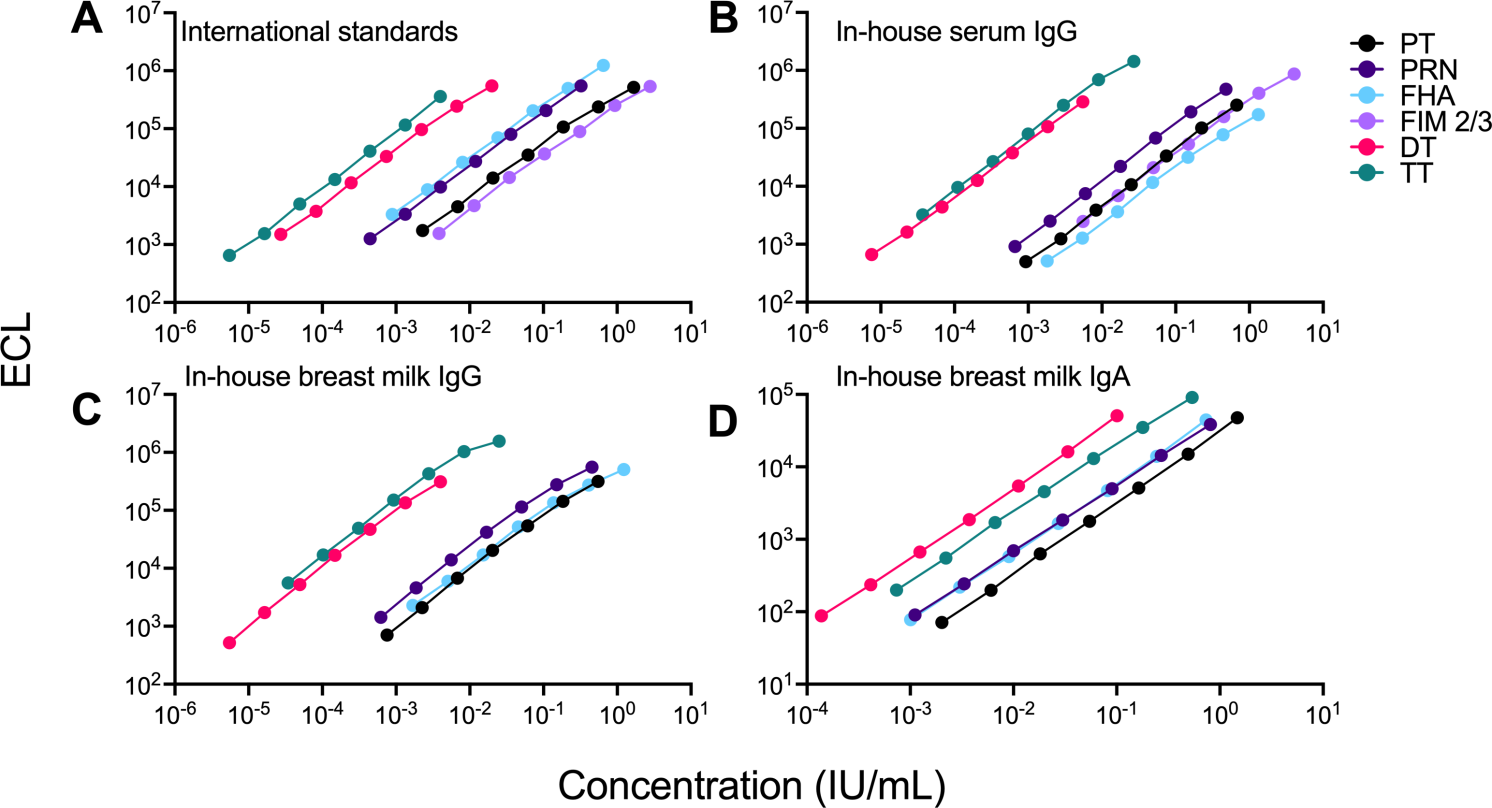
Dose–response curves for pertussis, diphtheria, and tetanus antibodies in the International and in-house standards measured by the multiplex assay. (**A**) WHO International Standard Pertussis (NIBSC 06/140), Tetanus Immunoglobulin (NIBSC TE-3), Diphtheria Antitoxin (NIBSC 10/262), and *B. pertussis* human serum (NIBSC 89/530); (**B**) in-house serum IgG standard; (**C**) in-house breast milk (BM) IgG standard; and (**D**) in-house BM IgA standard. Data depict mean electrochemiluminescence (ECL) units from duplicate wells versus antibody concentrations for PT (black), PRN (purple), FHA (light blue), FIM 2/3 (light purple), DT (pink), and TT (teal). Representative curves are shown. DT, diphtheria toxoid; FHA, filamentous hemagglutinin; FIM, fimbriae; PT, pertussis toxin; PRN, pertactin; TT, tetanus toxin. Curve parameters, given or assigned unitage, acceptance criteria, and detection limits are shown in Table 1

The optimal coating antigen concentrations were selected as those that produced the highest signal-to-noise ratio and produced ECL values for the standard curves well within the detection range: they were: 67 µg/mL for PT and DT; 33 µg/mL for TT, PRN, and FIM 2/3; and 15 µg/mL for FHA. The same criteria were used to select PBS + stabilizer + BSA as the optimal coating buffer. An in-house standard was established; this preparation was calibrated against the international standards for IgG concentration against all antigens and included in all assays for antibody quantification. Representative curves of ECL (signal) values versus antibody concentration for the International and in-house serum IgG standard are shown in Fig. 1A and B. Standard curve parameters: coefficient of determination (*R*^2^) and hillslope, as well as IgG geometric mean concentrations (GMCs) obtained from a total of 30 runs by two different operators are shown in Table 1. The IgG GMC against each antigen in the in-house standards ±20% was established as acceptable range (Table 1). Assay qualification results, including specificity, accuracy and precision, Lower Limit of Quantification (LLOQ), linearity and parallelism, matrix effect, and robustness are outlined in Table 2. The assay met NIAID/DMID qualification requirements for secondary endpoint antibody measurement.

**TABLE 1:** Standard curve parameters

Parameter	Antigen					
	PT	PRN	FHA	FIM 2/3	DT	TT
International WHO standard						
Coefficient of determination (*R*^2^)	0.99	0.99	0.99	0.99	0.99	0.99
Calculated line slope	0.97	0.96	0.94	0.89	1.01	0.99
GMC (IU/mL)	335	65	130	560	2.0	120
Acceptance criteria (±20% GMC)	268–402	52–78	104–156	448–672	1.6–2.4	96–144
Detection limits (IU/mL)						
Calculated low	0.00241	0.00047	0.00104	0.00429	0.00003	0.00001
Calculated high	1.680	0.325	0.650	2.8	0.020	0.004
In-house standard						
Coefficient of determination (*R*^2^)	0.99	0.99	0.99	0.99	0.99	0.99
Calculated line slope	0.99	0.98	0.98	0.97	0.98	0.98
GMC (IU/mL)	134.7	96.3	263.7	807.1	1.1	5.4
Acceptance criteria (±20% GMC)	107.8–161.6	77.1–115.6	211.0–316.5	645.6–1,452.7	0.9–1.3	4.3–6.5
Detection limits (IU/mL)						
Calculated low	0.00100	0.00086	0.00200	0.006	0.00095	0.00004
Calculated high	0.67	0.480	1.320	4.04	0.006	0.030
In-house standard breast milk IgG						
Coefficient of determination (*R*^2^)	1.00	1.00	1.00	-	1.00	0.98
Calculated line slope	0.97	0.97	0.98	-	1.00	1.09
GMC (IU/mL)	5.49	4.54	12.3	-	0.04	0.25
Acceptance criteria (±20% GMC)	4.39–6.59	3.63–5.45	9.84–14.76	-	0.03–0.05	0.20–0.30
Detection limits (IU/mL)						
Calculated low	0.00081	0.00065	0.00172	-	0.00001	0.00004
Calculated high	0.549	0.454	1.230	-	0.004	0.025
In-house standard breast milk IgA						
Coefficient of determination (*R*^2^)	0.99	0.99	0.99	-	0.99	0.99
Calculated line slope	1.09	0.97	1.06	-	1.09	0.99
GMC (IU/mL)	44.20	24.31	22.00	-	3.02	16.12
Acceptance criteria (±20% GMC)	35.36–53.04	19.45–29.17	17.60–26.40	-	2.42–3.62	12.90–19.34
Detection limits (IU/mL)						
Calculated low	0.00477	0.00180	0.00207	-	0.00033	0.00096
Calculated high	1.473	0.810	0.733	-	0.101	0.537

**TABLE 2:** Serum IgG assay qualification parameters

Parameter	Antigen					
	PT	PRN	FHA	FIM 2/3	DT	TT
Specificity (%)^*a*^						
PT	**88.4**	0.0	0.0	0.0	8.8	0.0
PRN	11.1	**98.1**	0.6	16.1	8.6	1.9
FHA	8.0	0.0	**95.2**	0.0	8.4	0.0
FIM 2/3	0.0	4.7	0.0	**98.9**	0.0	0.0
DT	9.4	5.0	6.7	5.7	**99.0**	4.6
TT	0.0	0.0	0.0	0.0	9.3	**99.3**
Relative accuracy and precision						
Mean bias (%RE)	−9.9	−3.7	3.2	−6.7	−8.3	−4.8
Repeatability (%CV)	8.6	4.9	9.3	7.4	5.8	9.6
Intermediate precision (%CV)	13.2	11.1	14.4	11.6	11.7	12.0
Limits of detection	0.001	0.0007	0.002	0.006	0.00001	0.00003
In-house standard (mean)						
Limits of quantitation (IU/mL)						
LLOQ (IU/mL)	1.00	0.70	2.00	6.00	0.01	0.04
Mean bias (%RE)	−14.3	−16.1	−15.2	−15.5	−9.2	−11.7
Repeatability (%CV)	10.2	6.20	7.78	7.20	12.20	9.56
Intermediate precision (%CV)	9.56	6.01	7.21	7.08	11.64	9.56
Linearity and dilutability						
Coefficient of determination	1.00	1.00	1.00	1.00	1.00	0.98–1.0
Regression line slope	1.04–1.07	0.96–0.99	0.97–1.00	0.94–1.01	0.91–1.00	0.85–0.973
Matrix effect						
Serum/plasma ratio	1.03	0.96	0.90	0.91	1.00	0.89
95% CI	0.99–1.07	0.91–1.02	0.79–1.01	0.81–1.01	0.97–1.03	0.79–0.99
Robustness						
75 min (IU/mL)^*b*^	61.4	63.9	232.4	326.4	0.6	3.8
60 min (IU/mL)^*c*^	60.0	60.7	254.8	313.9	0.5	3.6
*P* value	0.38	0.09	0.18	0.49	0.06	0.59
Concentration						
Mean of ratios *^b^*^*/c*^	1.027	1.043	0.893	1.058	1.043	0.992
SE	0.017	0.04	0.033	0.036	0.012	0.047

^*a*^ Bolded numbers represent % inhibition with matching assay antigen.

^*b*^ Antibody titers from assay using 75 min incubation.

^*c*^ Antibody titers from assay using 60 min incubation.

#### Parallelism

Slopes of dose-response regression curves from serially diluted serum samples spanning the assay range were compared with those of the in-house standard; no statistically significant differences were observed for any of the antigens tested (Fig. S1).

#### Dilutional linearity

Log transformed regression curves of calculated antibody concentration versus expected antibody concentrations for serially diluted serum and plasma samples showed excellent (linear) dose response for all antibody specificities (Fig. S2); coefficient of determination (*R*^2^) and regression line slopes (ranges) are shown in Table 2.

#### Specificity and sensitivity

Pre-incubation of immune sera with each of the target antigens inhibited ≥90% of specific antibody binding, whereas inhibition of non-specific antibody binding was ≤16% (Table 2). Assay specificity was therefore >90% for all antigens included in the assay. Assay sensitivity was confirmed through the determination of LLOQ levels for antibodies against each antigen (Table 2).

#### Accuracy and precision

Samples representing various antibody content (Hi, Mid, Low, and Very low) had a coefficient of variation (CV) <10% for measures of repeatability and <15% CV for immediate precision across all six antigens. Accuracy was confirmed at various levels of quantification. Mean bias, representing relative error of the calculated antibody concentration was <5%. Titers of mock samples with very low antibody content were well between 75% and 125% of the expected value.

#### Robustness

Increasing incubation time by 15 min resulted in higher in ECL values, which is expected, as raw ECL values may be impacted by assay conditions. However, the concentrations calculated through the in-house standard were not affected (*P* values > 0.05); the ratios of antibody concentrations determined in each condition fell within 95% CI.

### Multiplex assay for quantification of antibodies in breast milk

The multiplex assay developed for analysis of serum antibodies was adapted for determination of antibodies in breast milk. An in-house standard was calibrated against the same international standard preparations described above for serum assays. The in-house breast milk standard exhibited excellent linearity for all antibody specificities (Table 1); representative IgG and IgA dose-response curves against PT, PRN, FHA, DT, and TT are shown in Fig. 1C and D. Low- and high-titer positive controls were identified and included in the assay. A low titer control was used in lieu of a negative control due to the difficulty of obtaining negative samples; we attempted to deplete immunoglobulins to produce a negative control, but the procedure altered breast milk composition. The LLOQ values for the breast milk multiplex assay are indicated in Table 1. The assay met NIAID/DMID requirements for secondary analysis of IgG and IgA in the breast milk matrix.

### Determination of immune status in pregnant women

**Fig 2:**
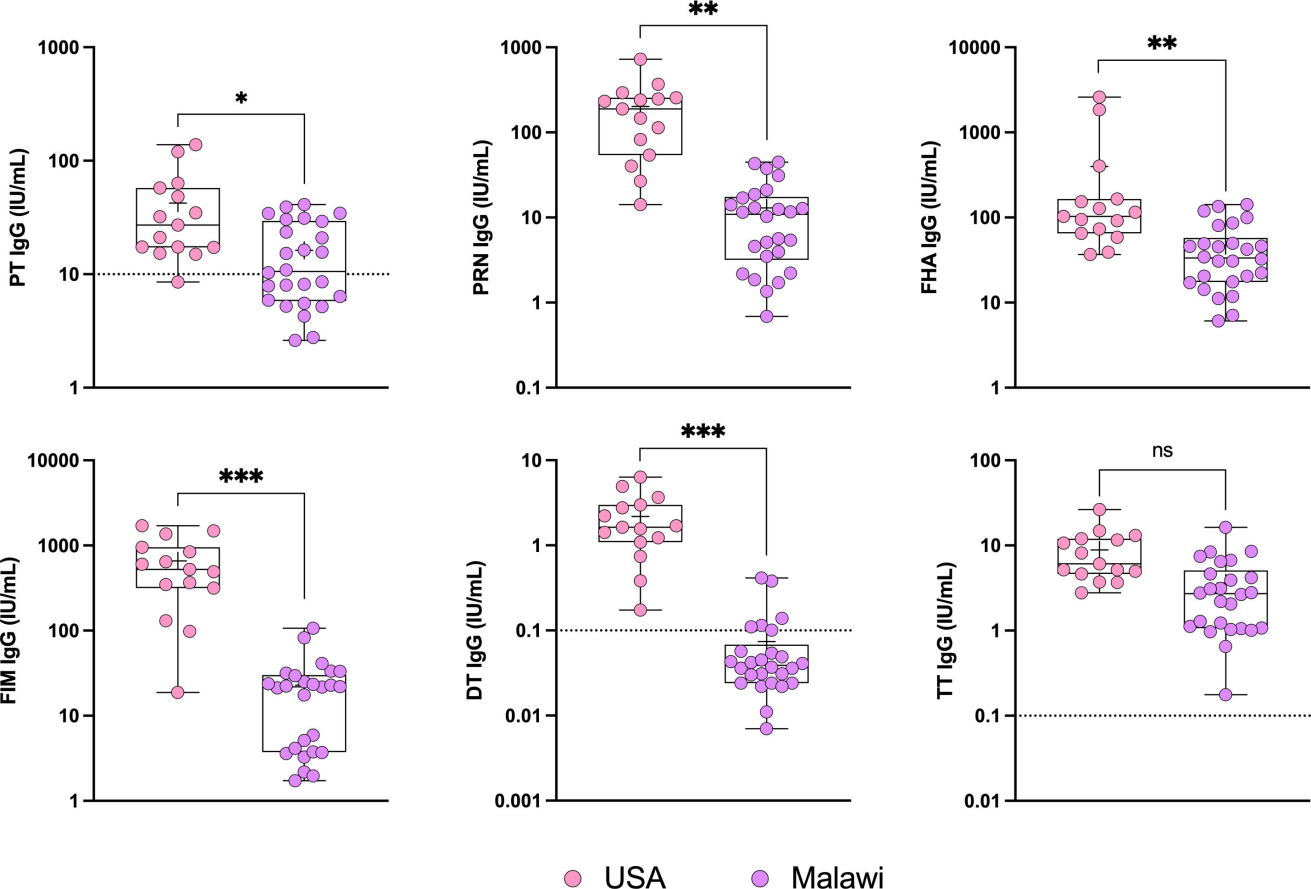
Serum IgG levels against PT, PRN, FHA, FIM 2/3, DT, and TT in pregnant women with different vaccination histories. Data represent antibody titers in women living in the United States and immunized with Tdap during pregnancy (*n* = 15), and women living in Malawi who received only tetanus vaccination (not Tdap or Td) during pregnancy (*n* = 26). Dotted lines represent protective thresholds. *P < 0.05, **P < 0.01, ***P < 0.001 by Kruskal-Wallis *H* test followed by the Dunn’s pairwise comparison with Sidák adjustment for multiple comparisons. DT, diphtheria toxoid; FHA, filamentous hemagglutinin; FIM, fimbriae; PRN, pertactin; PT, pertussis toxin; TT, tetanus toxin.

To confirm the utility of the multiplex assay and ability to discriminate humoral immunity in groups with different vaccination histories, we measured levels of pertussis, tetanus, and diphtheria antibodies in women living in the United States who receive Tdap during pregnancy and in women living in Malawi who receive only tetanus vaccine during pregnancy. Antibodies specific for PT, PRN, FHA, FIM 2/3, DT, and TT was determined in serum samples (Fig. 2).

We found that women living in the United States had higher antibody levels than those in mothers living in Malawi for all antigens tested except for TT; the difference between groups was significant with and without multiple comparison adjustment. Hence, the MSD multiplex assay was deemed proficient to distinguish serological status in groups with different immunization backgrounds.

### Longitudinal analysis of Tdap responses in post-partum women

**Fig 3:**
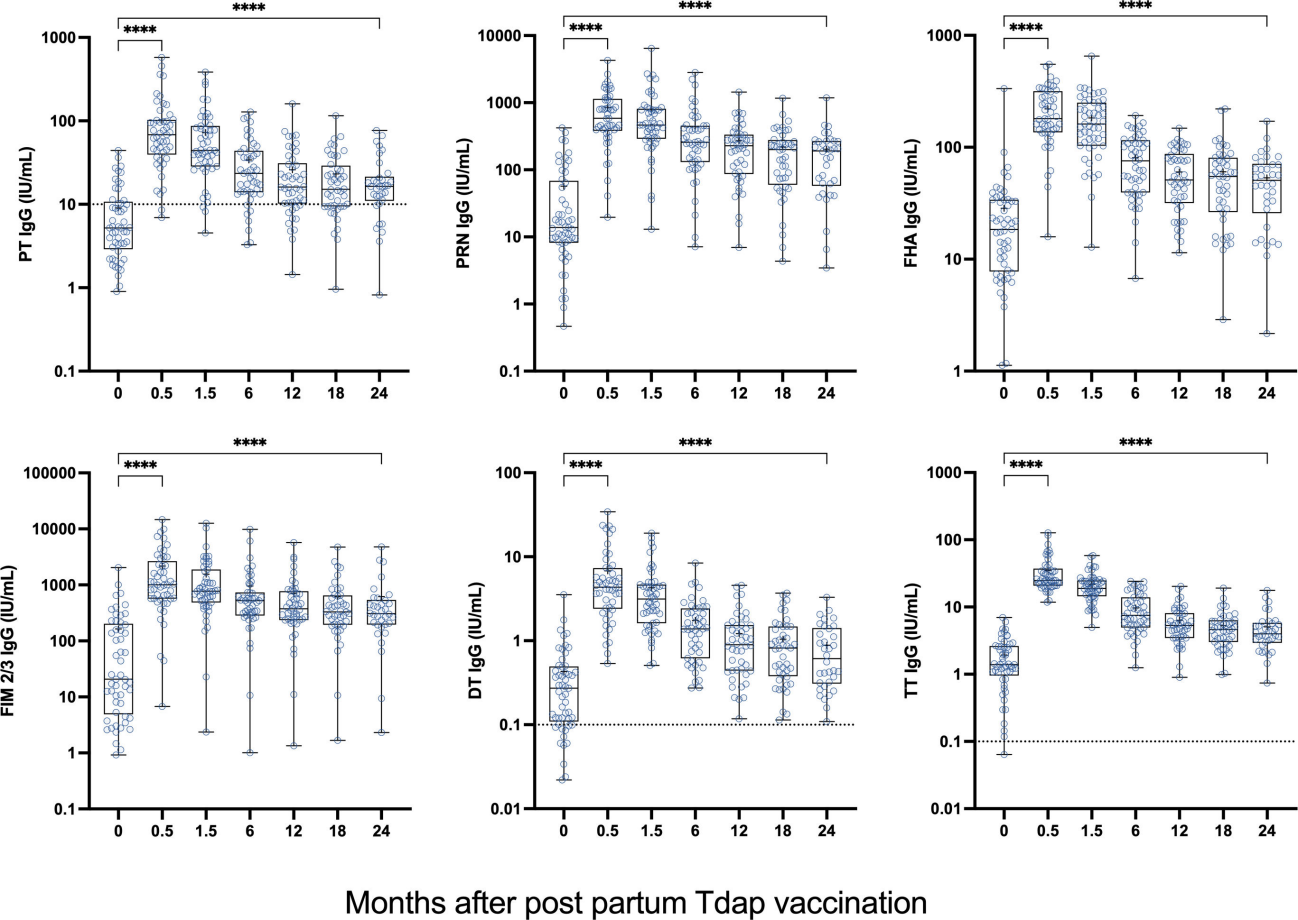
Serum IgG levels against PT, PRN, FHA, FIM 2/3, DT, and TT in post-partum women immunized with Adacel Tdap. Women were vaccinated 1–4 days after delivery. Data represent antibody titers before (0) and 0.5–24 months after post-partum Tdap vaccination. Fifty-three women provided samples at day 0; 34 remained in the study and provided blood at 24 months. Dotted lines represent protective thresholds, when available. ****P < 0.0001 by Wilcoxon signed-rank test. DT, diphtheria toxoid; FHA, filamentous hemagglutinin; FIM, fimbriae; PRN, pertactin; PT, pertussis toxin; TT, tetanus toxin.

Next, we examined antibody responses to Tdap in sera and breast milk from U.S. women vaccinated post-partum. We also evaluated the kinetics and persistence of antibodies in both compartments. Antibodies to all antigens increased rapidly following vaccination. Peak serum IgG responses were seen at the 2-week time point (Fig. 3). A decline was observed in subsequent blood draws; at 6 months, mean titers had decreased by half of their respective 2-week values. Although titers declined over time, they remained elevated above pre-vaccination levels for 24 months (the last blood draw). The individual trajectories of serum IgG responses for all Tdap antigens exhibited the same trend—a 2-week peak followed by a gradual decline (Fig. S3). Post-vaccination PT mean IgG levels exceeded 10 IU/mL (putative protective level) at all time points. Similarly, post-vaccination mean IgG titers against DT and TT were above 0.1 IU/mL and 1 IU/mL protective thresholds, respectively. Response rate (individuals with ≥4-fold rise in titers at either 0.5 or 1.5 months post-vaccination) was between 68% and 100%; the highest seroconversion rate (100%) was observed for TT, with other antigens following (68–91%) (Table 3).

**TABLE 3:** Vaccine response rate (%)^*a*^

Month post vaccination	PT	PRN	FHA	FIM 2/3	DT	TT
0.5	87.8	79.6	93.9	67.4	89.8	100
1.5	80.4	74.5	88.2	64.7	86.3	92.2
Max^*b*^	**88.7**	**81.1**	**90.6**	**67.9**	**88.7**	**100**

^*a*^ Response rate or percent seroconversion is defined as >4-fold rise in serum IgG titers over baseline at either 0.5 month (*n* = 49) or 1.5 months (*n* = 51) post-vaccination.

^*b*^ Based on the highest (max) response over the time points measured (*n* = 53).

**Fig 4:**
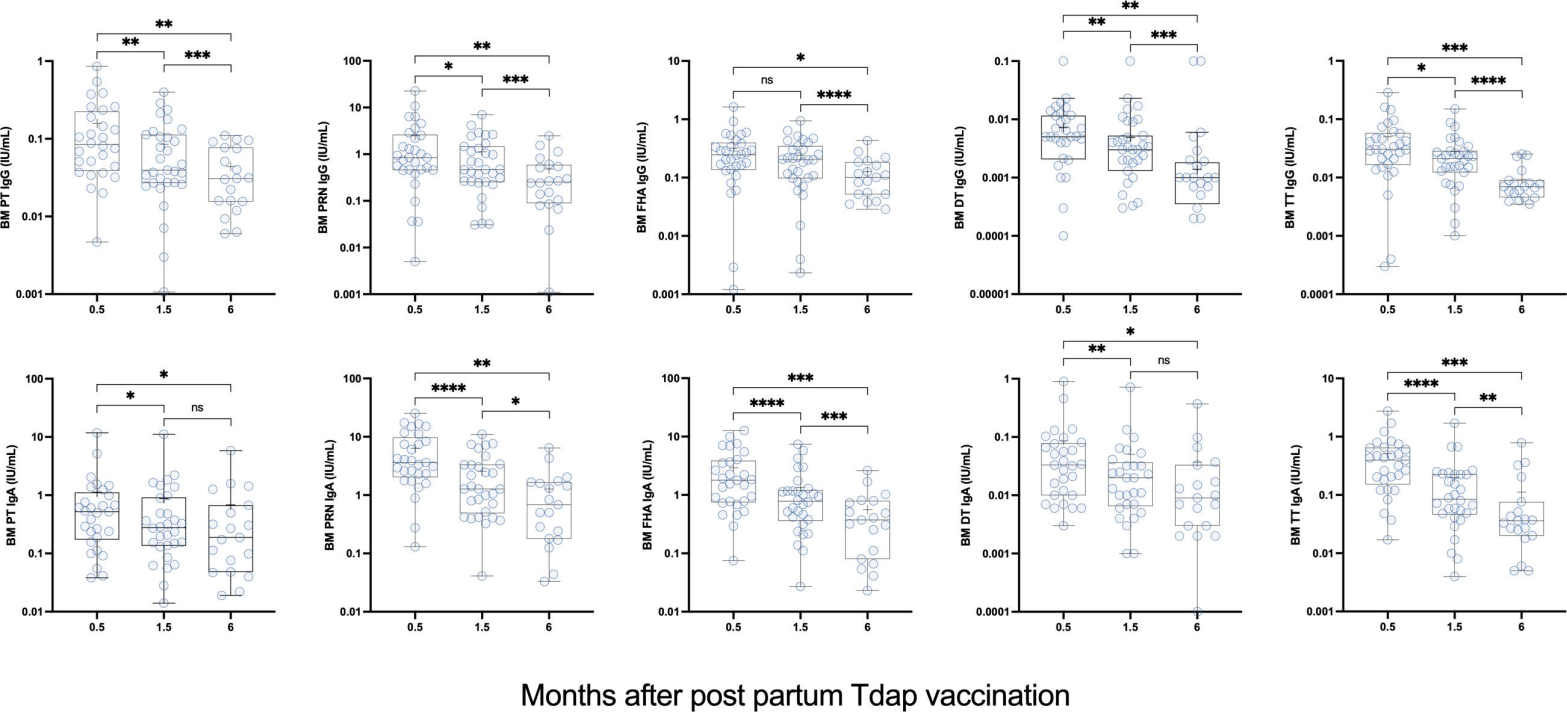
Breast milk IgG and IgA antibodies against PT, PRN, FHA, DT, and TT in post-partum women immunized Adacel Tdap. Data represent antibody titers 0.5–6 months after post-partum Tdap vaccination. Breast milk samples were available for testing from 18 women at 2 weeks, 31 women at 6 weeks, and 19 women at 6 months. *P < 0.05, **P < 0.01, ***P < 0.001, and ****P < 0.0001 by Wilcoxon signed-rank test. DT, diphtheria toxoid; FHA, filamentous hemagglutinin; FIM, fimbriae; ns, not significant; PRN, pertactin; PT, pertussis toxin; TT, tetanus toxin.

Vaccine-specific IgG and IgA were detected in breast milk from a subset of the same participants (Fig. 4). The highest antibody concentrations were detected 2 weeks post-vaccination (the first time point tested). Antibodies in breast milk decreased 1.5 months after vaccination and even further at the 6-month time point. For all antigens, breast milk IgG and IgA concentrations 6 months post-vaccination were significantly lower as compared with those at 2 weeks post-vaccination; (Fig. 4). The individual trajectories of breast milk IgG and IgA in post-partum Tdap vaccinated women replicate the 2-week peak response and subsequent decline (Fig. S4).

**Fig 5:**
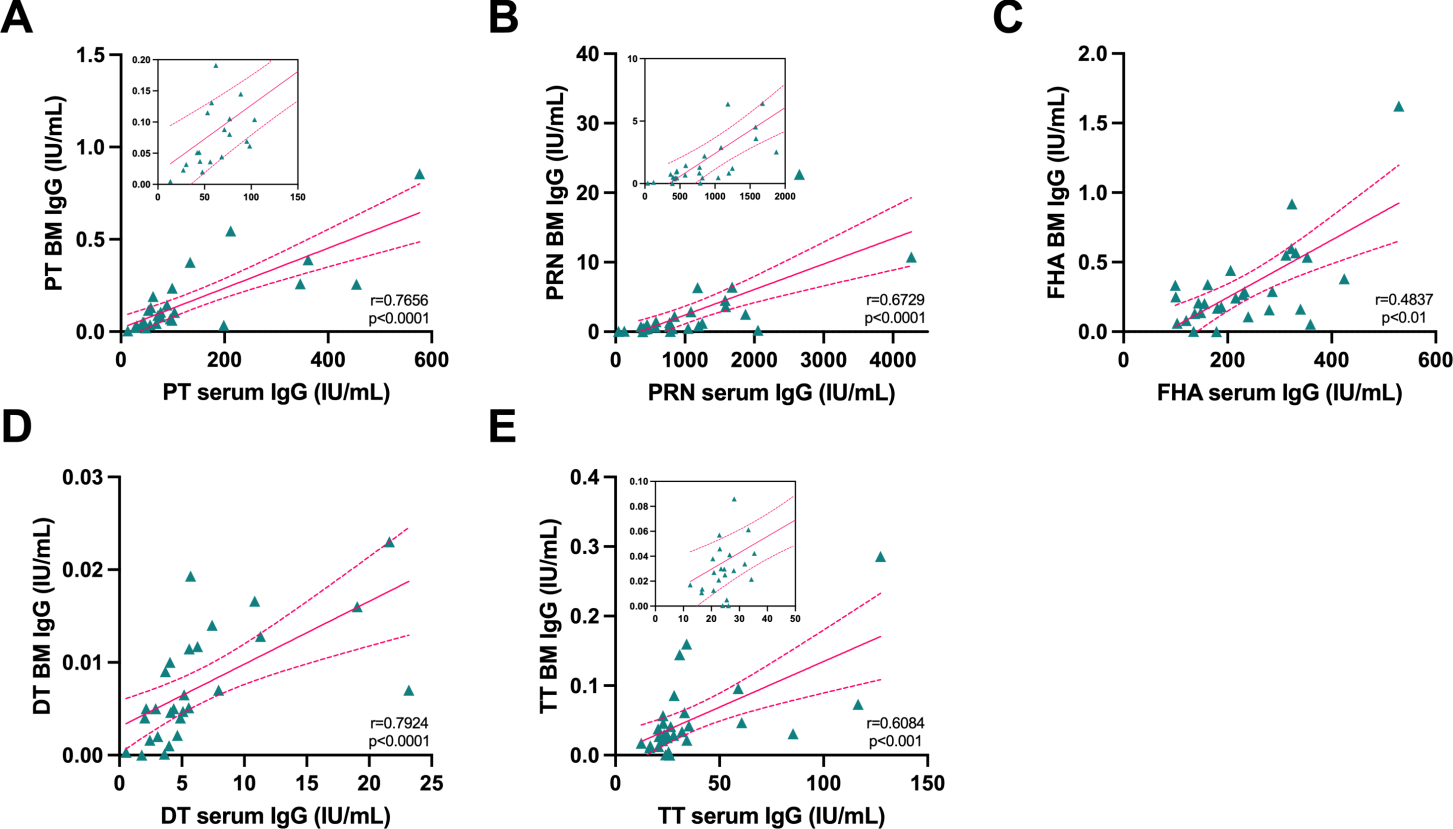
Association between serum and breast milk (BM) antibody levels in post-partum women immunized with Adacel Tdap. Correlation analysis of peak serum and breast milk IgG antibody titers (0.5 months post-partum) against Tdap antigens: (**A**) PT, (**B**) PRN, (**C**) FHA, (**D**) DT, and (**E**) TT. In-picture plots magnify data points and correlation at the lower end of the scale. DT, diphtheria toxoid; FHA, filamentous hemagglutinin; PRN, pertactin; PT, pertussis toxin; TT, tetanus toxin. *P* value and *r* = Spearman correlation are indicated.

IgG titers in breast milk were lower than those measured in serum. Notwithstanding, there was a strong correlation between peak IgG titers in both compartments (Fig. 5). Vaccine-specific IgG and IgA levels in breast milk were not significantly associated (data not shown). Further analyses are underway to dissect the structural features and anti-microbial function of breast milk antibodies.

## DISCUSSION

The determination of pertussis, tetanus, and diphtheria serological status and antibody responses to vaccination in the context of pregnancy and early infancy is important to discern the risk of infection and guide immunization strategies. Understanding the magnitude, breadth, and functional features of maternal antibodies is critical since it is the only element of adaptive immunity available to infants in the first months after birth (6, 7). New vaccines and approaches to enhance maternal immunity are being explored, and reliable and practical tools to facilitate their clinical evaluation are needed (8).

To support the analysis of pertussis, tetanus, and diphtheria immunity in the context of maternal-infant immunization, we have developed and qualified an MSD multiplex assay that allows simultaneous quantification of vaccine-induced antibodies, specifically, serum IgG directed against pertussis PT, PRN, FHA, FIM 2/3, as well as tetanus and diphtheria antitoxin. An in-house standard was established and assigned antibody concentrations (for all specificities) by calibration against available international standards. Antibody titers were calculated by backfitting ECL values into the in-house standard curve and reported in international (standard) units. Parallelism was demonstrated for the in-house standard and samples as well as excellent dilutional linearity.

The MSD assay had a wide dynamic range (spanning 3–6 logs) for all antigens, which allowed for high throughput testing of study samples at a single dilution, avoiding the need for repeats. Additional important analytical attributes of the MSD assay included its high sensitivity, specificity, accuracy, precision, and robustness (9). These analytical attributes were consistent with or generally superior to those reported in the literature for other assays (10–13).

Both serum and plasma could be tested interchangeably. The sample-sparing feature of our MSD assay (starting serum dilution: 1:1,000) makes it attractive for pediatric studies—particularly those involving infants. The assay high sensitivity (low LLOQ for all antigens) is advantageous for testing field serosurveys using easier to obtain samples (e.g., dry blood spots) or mucosal samples (oral fluid and nasosorption) that have lower antibody content than sera.

Tetanus, diphtheria, and pertussis serum antibody levels have traditionally been determined by ELISA, using either commercial or in-house assays (14). Commercial serologic assays, often designed for ease of use, lack sensitivity, precision, and accuracy for use in clinical vaccine evaluation. They do not provide information on critical reagents and analytical assay performance (15). Most of these assays are not calibrated against international standards. PT IgG detection kits developed for diagnostic use have limited range (usually centered around diagnostic cutoff), high variability and are not appropriate for precise antibody quantification (16, 17).

Multiplex assays for simultaneous measurement of pertussis, tetanus, and diphtheria antibodies have been previously described in the literature, all based on Luminex technology (10, 11, 18–21). The Luminex platform employs a mix of distinct fluorescent beads to which antigens are covalently attached; binding antibodies are detected through fluorescence intensity. An advantage of the MSD technology is that antigens do not require conjugation of vaccine antigens to beads, reducing potential impact on antibody reactivity. The use of manufactured antigen-printed plates (MSD system), as opposed to the manual coupling of antigens to beads (Luminex), reduces variation, and increases consistency. In addition, heterophilic antibodies in human sera may bind to the beads, increasing background signals (22). The MSD plate readout time is <1 min versus >30 min for Luminex; the reduced assay time enables high throughput testing. Higher sensitivity, accuracy, dynamic range, and lower matrix effect have been reported for the MSD platform across different applications (23–29). An advantage of Luminex remains the expandability for multiplexing in a single measurement.

Varghese et al. at Sanofi Pasteur reported the development and validation of an MSD multiplex assay for quantitative evaluation of pertussis (PT, FHA, FIM, and PRN) serum IgG for use in pertussis clinical vaccine development (30). Improving upon this work, our MSD assay was also qualified for quantification of tetanus and diphtheria antibodies, incorporates WHO international standards as calibrators and reports titers in IU/mL—as recommended (31) and was successfully applied to a new matrix (i.e., breast milk). The Varghese study included a comparison of the MSD multiplex with commercial PT antibody assay kits, and showed the MSD platform’s superior linearity, accuracy, and detection range, and discrimination of pre- and post-vaccination samples. The MSD platform has also been validated for quantification of antibodies against a variety of other pathogens (32), most recently SARS-CoV-2 (33, 34) and was used in the clinical evaluation of the COVID-19 vaccine sponsored by the US government (35).

Our multiplex assay described was initially developed to generate serological primary endpoint data for an NIH sponsored Phase II clinical study that evaluated Tdap immunization of pregnant women in Mali and the impact of this intervention on vaccine responses in the infants (36). Following NIAID/DMID guidelines (37), the assay was fully qualified, and adequate performance and suitability for its intended purpose were demonstrated.

In preparation for the serological analyses of the clinical samples from women in Mali, we conducted a feasibility study whereby IgG levels specific to Tdap antigens were determined in convenience serum samples from pregnant women living in the United States and pregnant women living in Malawi. The former group, but not the latter, received Tdap during pregnancy. Antibodies to all pertussis antigens were consistently higher in the US women. Diphtheria antibodies were also elevated. In contrast, no significant differences between the two cohorts were seen in the level of tetanus antitoxin, because tetanus vaccines are routinely administered to pregnant women living in sub-Saharan Africa. Thus, the multiplex assay successfully differentiated the two groups based on serological status, with antibody titers matching local vaccination practices.

The MSD assay was further successfully adapted for quantification of antibodies in breast milk. Antibodies in breast milk provide protection during the early months of life and it is therefore important to understand their magnitude and longevity (38). There is a paucity of information on pertussis, tetanus, and diphtheria humoral immunity in lactating women.

The MSD multiplex technology was aptly applied to determine Tdap responses in serum and breast milk from US women vaccinated post-partum. A longitudinal analysis of antibody titers enabled us to interrogate duration of immunity post vaccination. Our kinetic analysis revealed a sharp increase in serum IgG responses to all vaccine antigens 2 weeks post-vaccination with 68–100% seroconversion rate. The timing of peak responses in our study is consistent with serological data reported by Halperin et al. in Canadian women vaccinated post-partum (2). In our post-partum cohort, PT IgG titers post-vaccination were above the putative protective threshold of 10 IU/mL (39), and TT and DT titers were above the 0.1 IU/mL protective threshold (40). A steady decline in antibodies was observed thereafter; a decrease in circulating antibody levels is expected and consistent with the maintenance of immunity via memory B cells. Despite this decline, serum IgG titers to all vaccine antigens remained elevated above baseline (and exceeding protection threshold levels) up to 24 months post-vaccination, the last time point evaluated, and this was true for all antigens.

Vaccine-specific IgG and IgA were detected in breast milk; the highest titers were observed closest to the time of vaccination (2 weeks later). Although titers declined thereafter, they persisted for at least 6 months. An important feature of our cohort is the extended sampling; previous longitudinal analyses of Tdap antibodies in breast milk spanned only 8–12 weeks (2–4). These studies also report waning of antibodies over time. The decline in Tdap immunity observed in maternal milk is consistent with the lessening of immunity in circulation. The composition of breast milk varies through the lactation period further impacting antibody content and availability (41).

Despite the difference in magnitude, the vaccine-specific IgG content in serum and breast milk—against all antigens, was strongly correlated. A positive linear relationship between breast milk and serum PT IgG had been reported in women vaccinated during pregnancy (4). Our analysis extended the correlative analysis of serum versus breast milk IgG to other specificities: tetanus, diphtheria, and other pertussis antigens (FHA and PRN) included in Tdap. The strong concordance between serum and breast milk IgG titers is consistent with the notion that maternal milk contains blood derived IgG (transported via the neonatal Fc receptor or via transudation) (42, 43), whereas IgA is produced locally by plasma blasts in the mammary gland and secreted through epithelial cells. Close agreement between blood and breast milk IgG has been reported in the context of HIV infection and COVID-19 vaccination (44, 45). Our results confirm that immunization with Tdap post-partum results in abundant and lasting vaccine-specific antibodies in the breast milk that will benefit the infants via lactation.

In summary, we report the development and qualification of a multiplex assay for quantitative analysis of pertussis, tetanus, and diphtheria antibodies that is specific, sensitive, accurate, reproducible, and suitable for the study of seroprevalence and the evaluation of immune responses to vaccination. This assay can be applied to the analysis of antibodies in serum and breast milk and is a reliable and practical tool for producing clinical study endpoints.

## MATERIALS AND METHODS

### Antigens and standards

PT, PRN, and FIM 2/3 were obtained from List Biological Laboratories (Campbell, CA). FHA was obtained from Enzo Life Sciences (Farmingdale, NY). DT and TT were obtained from Statens Serum Institut (Copenhagen, Denmark) and from the National Institute for Biological Standards and Control (NIBSC, Hertfordshire, UK), respectively.

The World Health Organization (WHO) International Standard Pertussis human antiserum (NIBSC 06/140) was used as a standard/calibrator for measurement of PT-, FHA-, and PRN-specific antibodies. *Bordetella pertussis* human serum (NIBSC 89/530) was used as calibrator to measure FIM 2/3-specific antibodies. WHO International Standards for Tetanus Immunoglobulin, Human (NIBSC TE-3), and Diphtheria Antitoxin Human (NIBSC 10/262) were used to quantify TT- and DT-specific antibodies, respectively. An in-house serum IgG standard (high-titer pooled sera) was prepared from a pool of six Tdap immunized donors, calibrated against the international standards (as described below), and used in routine testing. A separate high-titer serum sample served as positive control. Human serum minus IgA/IgM/IgG (Sigma-Aldrich, St. Louis, MO) was used as negative control. Two in-house breast milk standards (BM-IgG and BM-IgA) were prepared, calibrated against the international standards, and used in routine assays. Breast milk controls included high-titer and low-titer breast milk pools from five to eight TdaP immunized donors.

### Clinical samples

Serum and breast milk samples from the University of Maryland, Baltimore (UMB), blood donor protocol CVD-2000 (*n* = 6) were used to produce the in-house standard and controls. Breast milk samples were processed to obtain whey fraction by 2 rounds of centrifugation at 3,000 × *g* for 10 min at RT using a tabletop centrifuge, discarding the upper fat layer using a sterile cotton swab and collecting middle whey solution, avoiding particulate pellet.

Sera and breast milk were obtained from the Division of Microbiology and Infectious Diseases (DMID) study 11-0035 “Tdap vaccine in post-partum women” (NCT01711645). In this study, a single intramuscular (IM) 0.5 mL dose of Adacel was administered to healthy 1–4 days post-partum women who had delivered healthy full-term infants (18–45 years of age) at the time of hospital discharge. Adacel is a four-component vaccine that contains TT (5 Lf), DT (2 Lf), and pertussis antigens: detoxified PT (2.5 µg), FHA (5 µg), PRN (3 µg), and FIM 2/3 (5 μg). Sera was available from 53 mothers at enrollment (vaccination), 49 at 2 weeks, 51 at 6 weeks, 47 at 6 and 46 at 12 months, 44 at 18 months, and 34 at 24 months; ultimately, sera from 31 women were available at all time points. Breast milk samples were available from 28 mothers at 2 weeks, 31 at 6 weeks, and 19 at 6 months post-vaccination; breast milk from 15 women were available at all time points. Samples were missing from women who were lost to follow-up, withdrew voluntarily or were withdrawn from the study for planned or actual receipt of another dose of Tdap prior to study completion, or due to insufficient volume.

Convenience serum samples were also obtained from US women who had received Tdap during pregnancy through UMB blood donor protocol CVD-2000 (*n* = 15) and from a cohort of mothers living in Malawi (*n* = 26). Study participants from Malawi were recruited between January and November 2016 as part of a longitudinal malaria surveillance study at Mfera Health Clinic in Chikwawa, Malawi (46). Healthy pregnant women who were HIV seronegative were enrolled either at the prenatal clinic visit or during their hospital delivery stay. The Malawi mothers—all except for one would have received pertussis-, tetanus- and diphtheria-vaccines during childhood as part of the Expanded Programme on Immunization (EPI), which was launched in Malawi in 1979 (47) and tetanus immunization (not Tdap or Td) as standard antenatal care at the time of enrollment (48).

### MSD multiplex assay

#### Selection of optimal conditions and establishment of the assay

The MSD plate optimization package was used to define optimal antigen coating conditions; three concentrations (33, 67, and 130 µg/mL) of each antigen and two proprietary coating buffers (PBS + stabilizer or PBS + stabilizer + bovine serum albumin [BSA]) were tested. The WHO International Standards for Pertussis Anti-Serum (PT, FHA, and PRN), Tetanus Immunoglobulin (TT) and Diphtheria Antitoxin (DT), NIBSC *Bordetella pertussis* reference material (FIM 2/3), and negative control sera were included in each run during the selection of assay conditions. The assay procedures were established following the manufacturer’s recommendations. Briefly, plates were allowed to equilibrate for 15–30 min at room temperature (RT) and blocked with 150 µL of 10% non-fat dry milk in PBS-0.05% Tween 20 (assay diluent) for 1 h at room temperature. Plates were subsequently washed six times with 150 µL of PBS containing 0.05% Tween 20 and incubated for 1 h at RT with 50 µL of the standards, controls, and samples (all diluted in assay diluent), in duplicate. Wells with diluent buffer alone were included as blanks. During incubations, plates were placed on orbital plate shaker at 400 rpm. Plates were washed again as described above, and 50 µL of a 1.0 µg/mL solution of detection antibody (SULFO-TAG labeled goat anti-human IgG) were added and plates incubated for 1 h at RT in orbital plate shaker. Plates were washed as described above, and 150 µL of MSD Gold Read Buffer was added. Electrochemiluminescence (ECL) values were measured on the MSD QuickPlex SQ 120 reader within 20 min. Data were analyzed using MSD Discovery Workbench Version 4.0 (MSD, Rockville, MD). For selection of optimal coating conditions, ECL values obtained from international standards were divided by ECL signals of blanks to generate signal-to-noise ratios. Dose-response curves were generated for each of the standards using a 4-parameter logistic fit (4PL), with ECL values for blanks automatically subtracted. Curve fit statistics (*R*^2^) and plot of standards were produced by the MSD Workbench software. An in-house standard (described above) was included in subsequent assays, spanning seven 3-fold dilutions, starting at 1:200. The IgG or IgA concentrations for all antigens in the in-house standards were determined by comparison with the WHO international standards and NIBSC *Bordetella pertussis* reference material backfitting ECL signals to the 4PL standard curves. Positive and negative controls (described above) were also included in all assays.

Clinical serum samples were tested at 1:1,000 dilution, and at a higher dilution if the ECL did not fall within the assay detection range. Antibody concentrations in study samples and controls were determined by interpolation of the ECL from the 4PL regression of the in-house standard using MSD Workbench software. Units are reported in IU/mL.

#### Assay qualification for quantification of antibodies in serum

Plates were custom printed by MSD at the selected concentrations and stored at 4°C until use; the same batch of plates was used for all assay qualification experiments. The following assay performance features were investigated.

##### Linearity of calibrated standards

Linear regression curves were fitted for the International and in-house serum or breast milk standards. The Pertussis Antiserum (Human) WHO International Standard (for PT, FHA, and PRN) was tested starting at a 1:200 dilution, corresponding to 1.675 IU/mL of PT IgG, 0.65 IU/mL of FHA IgG, and 0.325 IU/mL of PRN IgG. The anti-*Bordetella pertussis* serum (Human) NIBSC Reference Material was also tested at a 1:200 starting dilution corresponding to an FIM 2/3 IgG concentration of 2.8 IU/mL. The WHO International Standard for Tetanus Immunoglobulin (Human) was tested starting at a 1:30,000 dilution, corresponding to 0.004 IU/mL of TT IgG, and WHO International Standard for Diphtheria Antitoxin Human was tested starting at 1:100 dilution, corresponding to an initial concentration of 0.02 IU/mL for DT IgG. The serum in-house IgG standard as well as the human breast milk IgG and IgA standard were tested as described in the serum and breast milk assay sections. Curves spanned seven dilutions.

##### Parallelism

Assay parallelism was assessed by comparing the slopes of linear regression curves of antibody concentration vs dilution factor (both log-transformed) of sera from two different donors with the in-house standard curve for each antibody specificity.

##### Assay linearity

To evaluate assay linearity, paired sera and plasma from two different immune donors with assigned antibody concentrations were tested at multiple serial threefold dilutions spanning the range of the assay. Antibody concentrations were calculated by interpolating ECL values from the in-house standard curves as described above. To assess dose-response linearity, the calculated antibody concentration for each sample dilution was plotted against the expected antibody concentration (both log-transformed), for all antibody specificities.

##### Specificity

To demonstrate assay specificity, antigen binding inhibition experiments were conducted by pre-incubating the in-house serum standard at 1:500 with 2.5 µg/mL of antigen in sample diluent, an excess amount of each of the assay antigens (PT, PRN, FHA, FIM 2/3, DT, and TT) and then testing adsorbed and non-adsorbed preparations. ECL values were recorded, and the percentage of inhibition was determined for both homologous and heterologous antigens as follows: 100 – (adsorbed sample ECL/non-adsorbed sample ECL × 100).

##### Accuracy and precision

Accuracy and precision were assessed by testing the in-house standard neat and “mock” samples prepared by pre-diluting the in-house serum standard (GMC of IgG against each antigen is listed in Table 2) with negative serum as follows: Hi (neat sera); Mid (1:2), Low (1:8), and very low (1:32) and even lower (1:64 and 1:128). These samples were tested in five plates and six independent tests per plate, which generated *n* = 30 datapoints for each dilution level for calculation of % coefficient of variation (CV) intra (repeatability) and %CV inter assay (intermediate precision) were calculated. In addition, mean bias measured by relative error percentage (%RE), was calculated (relative accuracy = calculated concentration/nominal concentration × 100) to determine accuracy or trueness (49). A similar analysis was conducted in further diluted mock samples (1:16,000–64,000) to confirm accuracy at lower levels.

##### Assay quantification limits

The LLOQ of the serum IgG assay was established as the lowest antibody concentration that could be quantified with acceptable accuracy and precision in the established assay conditions. It was calculated as the lower limit of detection (background + 2.5 standard deviations) calculated by the MSD Discovery Workbench software for the in-house standard curve multiplied by the dilution recommended for the assay. Relative accuracy and precision around the LLOQ were determined by testing mock low-level samples diluted in negative serum, over multiple days and in different plates. The ULOQ was also determined, although samples with antibody levels above ULOQ are further diluted to reach assay range.

##### Robustness

Assay robustness was determined by modifying incubation times. A panel of six samples (Hi, Mid, Low, and Very Low, and two additional serum samples) were tested using the established 60 min versus 75 min incubations for all assay steps (block, primary antibody, and secondary antibody), and ECL and antibody concentrations were compared.

### Multiplex assay for quantification of antibodies in breast milk

The assay was conducted as described above with the following modifications: the in-house breast milk standards (BM-IgA or BM-IgG) were included in each plate spanning seven 3-fold dilutions, starting at a 1:30 for IgA and at 1:10 for IgG, both in sample diluent. A high-titer positive control, low-titer control, and unknown breast milk samples were tested at 1:30 and diluted accordingly if the sample did not fall within detection ranges. Two breast milk controls were generated, a low-titer control prepared from a pool of eight low-titer human breast milk samples obtained 6 months post-partum from Tdap-vaccinated women, and a high-titer positive control prepared from a pool of five positive high-titer human breast milk samples obtained 2 weeks post-partum from Tdap-vaccinated women. The log-transformed IgG and IgA concentrations were plotted versus log-transformed dilution factor for each antibody specificity to assess assay linearity and parallelism.

### Quality control

The following system suitability criteria were established to determine whether assays were of acceptable quality. Goodness of fit (*R*^2^) for each standard curve must be ≥0.98. In addition, the percent recovery for all points within the linear portion of the standard curve must be between 80% and 120%. The mean of the blanks for each assay on each plate must be <200 ECL signal. The adjusted ECL signal (ECL for the blank subtracted) for negative control (if not within acceptable range) must be <500. The ECL signal and concentration for positive and negative control samples must be within the acceptable ranges determined during qualification. The maximum acceptable variation (%CV) between duplicate signals obtained for assay controls/reference standards is 25%. The %CV between duplicate wells for a sample is 25%. If these conditions were not met, the assay was repeated. In addition to serving as criteria for assay acceptance, these three parameters (i.e., standard curve goodness of fit, recovery, and positive control antibody concentration) are evaluated for each new lot of MSD printed plates prior to their use for testing of study samples. Requalification and bridging data were required if essential reagents were changed.

### Statistical analysis

Intra and inter-assay %CV were calculated based on a one-way analysis of variance, as previously described (49). Comparison of antibody levels among groups (Fig. 2) was performed using the Kruskal-Wallis *H* test followed by the Dunn’s pairwise comparison with Sidák adjustment for multiple comparisons as appropriate. Pre- and post-vaccine serum IgG responses: baseline versus 0.5 months post-partum (peak response time point) and versus 24 months post-partum (last time point) were compared using Wilcoxon signed-rank test. The number and proportion of responders (fourfold increase over baseline) were calculated at 0.5 months and 1.5 months, respectively. Additionally, the number and proportion of subjects who ever had a fourfold increase at 0.5 months or 1.5 months were calculated for each antibody of interest. The Wilcoxon signed-rank test was used to pairwise compare breast milk antibody levels at 0.5, 1.5, and 6 months post-partum. Correlation of serum and breast milk IgG antibodies were assessed using Spearman’s rank correlation. Statistical significance was set at *P* < 0.05 and all statistical analyses were conducted using GraphPad Prism Version 9.0 (GraphPad Software, San Diego, CA) or Stata/SE Version 17 (StataCorp, College Station, TX).

## SUPPLEMENTAL MATERIAL

Supplemental Material msphere.00527-23-s0001.pdf Fig. S1 to S4.

ASM does not own the copyrights to Supplemental Material that may be linked to, or accessed through, an article. The authors have granted ASM a non-exclusive, world-wide license to publish the Supplemental Material files. Please contact the corresponding author directly for reuse.
