# Supplementary material for: Quantitative analysis of pertussis, tetanus, and diphtheria antibodies in sera and breast milk from Tdap vaccinated women using a qualified multiplex assay
Source: mSphere. 2024 Mar 18;9(4):e00527-23. doi: 10.1128/msphere.00527-23 (PMC11036809; doi:10.1128/msphere.00527-23)
Supplement: Supplemental Material — Fig. S1 to S4. [file msphere.00527-23-s0001.pdf]

Supplemental Material

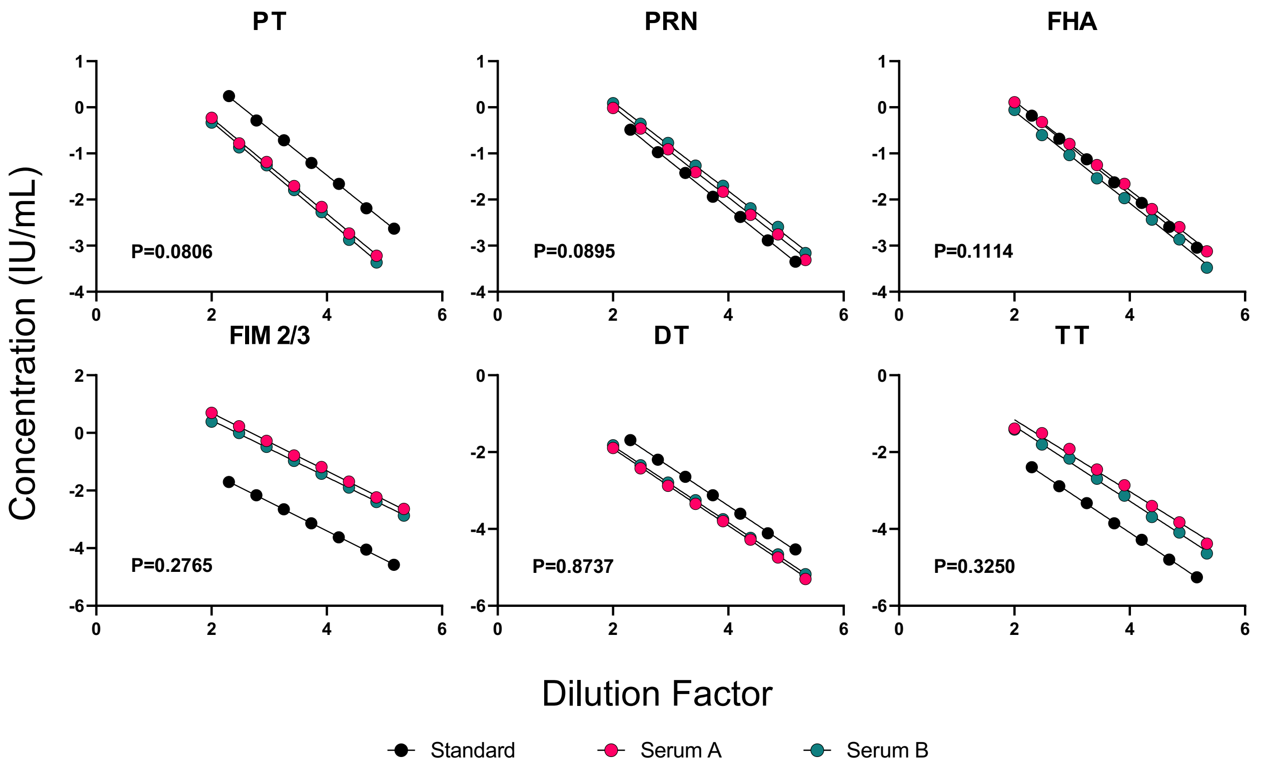

Fig S1. **Parallelism**. The in-house serum IgG standard (black) and serum samples from two different donors, Serum A (pink) and B (teal) tested in the MSD assay, in serial 3-fold dilutions. The concentration of antibodies (y axis) against each antigen (for each dilution tested) was determined and plotted against dilution factors (x-axis); both concentrations and sample dilution factors were log transformed. Linear regression curves are shown. P values (two-tailed) from the test of the null hypothesis that the slopes are identical are shown. P>0.05 indicates slopes for the in-house standard and serum samples are not statistically different.

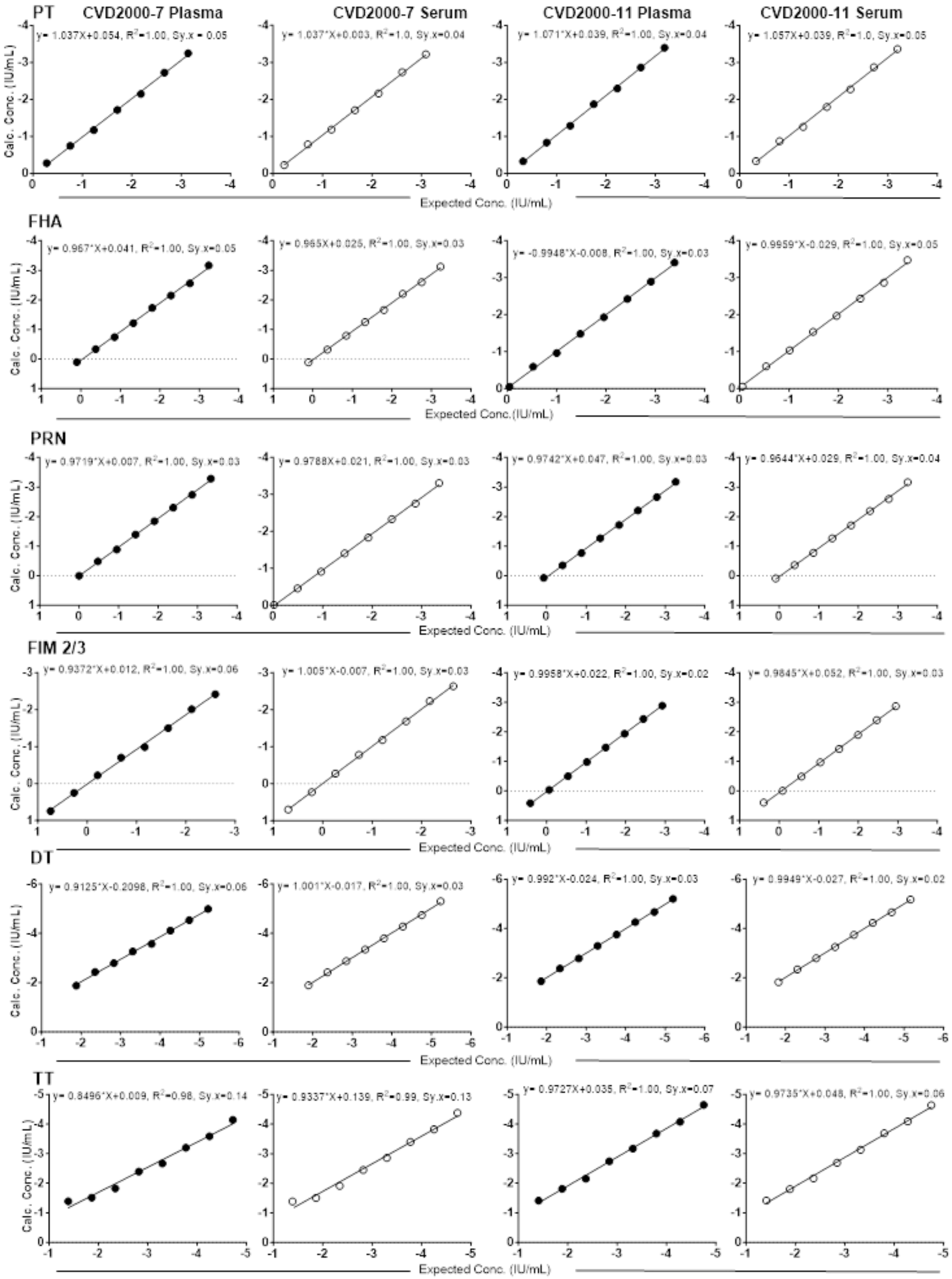

Fig S2. **Dilutional linearity**. Sera (black) and plasma (white circles) with known antibody concentrations were tested tested in the MSD assay, in serial 3-fold dilutions. The concentration of antibodies calculated for for each dilution tested (y axis) was plotted against the expected concentration (x-axis); both were log transformed. Linear regression curves, coefficient of determination (R^2) and standard error of the estimate (Sy.x) are shown.

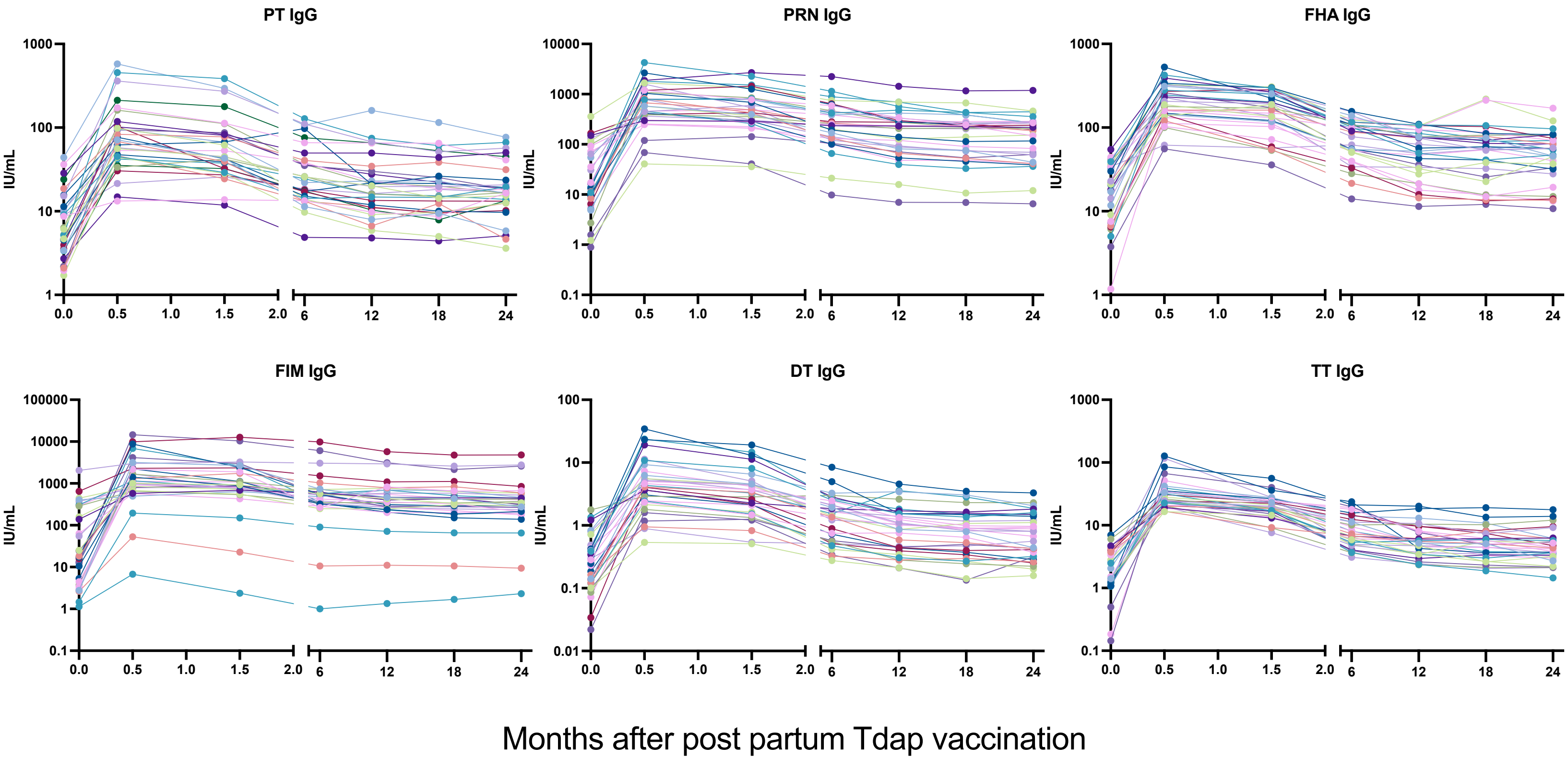

Fig S3. Individual trajectory of serum IgG responses to Tdap antigens. Lines represent antibody levels over time for individual subjects who received Tdap (Adacel) post-partum. Data represent titers from 31 women with complete samples from vaccination to month 24 . The kinetics of responses was similar for all individuals and involved a peak for all vaccine antigens 2 weeks post vaccination and subsequent antibody decline.

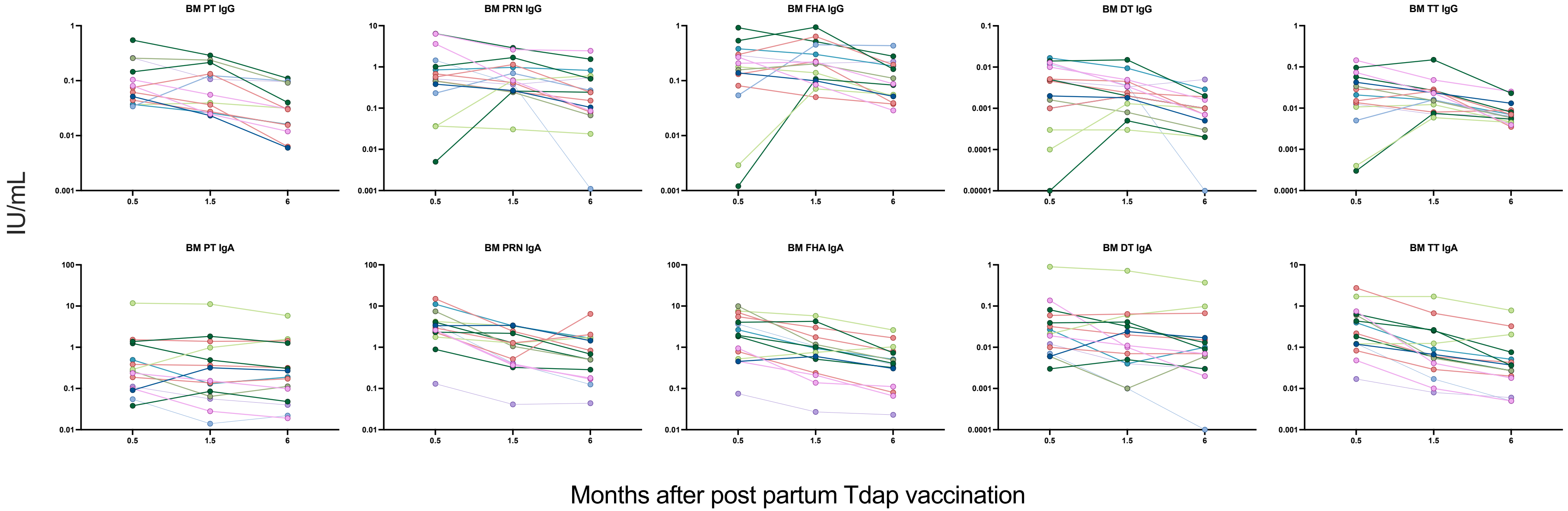

Fig S4. Individual trajectory of IgG and IgA antibodies to Tdap in breast milk (BM). Lines represent antibody levels over time from women who received Tdap (Adacel) post-partum. Data represent titers from 13-16 women with complete BM samples from 2 weeks to 6 months post vaccination vaccination.
